# Supplementary material for: RNA-seq Based Transcriptome Analysis of the Anti-Obesity Effect of Green Tea Extract Using Zebrafish Obesity Models
Source: Molecules. 2019 Sep 6;24(18):3256. doi: 10.3390/molecules24183256 (PMC6767142; doi:10.3390/molecules24183256)

Supplementary Materials

Table S1. Protein expression targets regulated by GTE in GTE-treated OF zebrafish compared with those of OF zebrafish.

Name	Total # of Neighbors	# of Measured Neighbors	Gene Set Seed	Measured Neighbors	Median change	p-value
Protein regulators of keratin	158	5	keratin	CEBPA;KRT8;KRT15;DLX3;RARG	-12071.127	0.006
Protein regulators of recombinase	135	5	recombinase	COL1A2;MYL1;COL1A1;KLF5;CKM	-5.329	0.014
Protein regulators of ADAMTS5	102	5	ADAMTS5	CEBPD;PRKCE;IHH;RARG;WISP3	-2602.579	0.016
Protein regulators of S100A8	89	5	S100A8	PLAU;CEBPA;PTGER4;KLF5;RARG	-5.529	0.019
Protein regulators of NR4A1	187	6	NR4A1	NKX6-1;HTR2A;CEBPA;NR1D1;PTGER4;RARG	3.478	0.023
Protein regulators of PAX6	211	6	PAX6	CHRD;MID1;ENTPD2;SIX3;BARHL2;KCNJ6	-7.108	0.023
Protein regulators of ITGA5	123	5	ITGA5	CEBPA;HSP90B1;ITGB4;OLFM4;DLX3	-11.961	0.025
Protein regulators of COL10A1	198	8	COL10A1	CEBPA;SMAD1;CBFB;CHRD;IHH;SMPD3;RARG;WISP3	-3.177	0.032
Protein regulators of WNT1	107	7	WNT1	NKX6-1;ONECUT1;WLS;FST;GBX2;ZIC1;SIX3	-1311.806	0.036
Protein regulators of NEUROD1	151	5	NEUROD1	NKX6-1;NCAM1;NR4A1;ZIC1;FHL1	-2.885	0.046
Protein regulators of KLF4	264	9	KLF4	ITLN1;CEBPA;KCTD12;IGFBP5;ZNF750;KLF5;DIRAS1;GBX2;AGBL4	-3.673	0.047
Protein regulators of MTOR	207	8	MTOR	CEBPD;HMGB1;HSP90B1;BZW2;NUAK2;AMPD1;GRPR;WISP3	-4.025	0.048

Table S2. Gene expression ratios in WNT/β-catenin signaling pathway in GTE-treated OF zebrafish compared to those in OF control zebrafish.

Gene symbol	Description	GTE vs. OF (log ratio)
WNT	Wingless-type MMTV integration site family	9.985
FZD	Frizzled family	9.914
SMARCA4	SWI/SNF related, matrix associated, actin dependent regulator of chromatin, subfamily a, member 4	-0.557
AXIN1	Axin 1	-0.298
CTNNB1	Catenin beta 1	-0.181
APC	APC, WNT signaling pathway regulator	-0.091
WIF1	WNT inhibitory factor 1	-0.622
LRP5	LDL receptor related protein 5	0.566
LRP6	LDL receptor related protein 6	-0.277
SOST	Sclerostin	-2.786
GBP1	Guanylate binding protein 1	-4.403
GSK3B	Glycogen synthase kinase 3 beta	0.717
CSNK1E	Casein kinase 1 epsilon	-2.102
CREBBP	CREB binding protein	-0.565
CSNK2A1	Casein kinase 2 alpha 1	-3.597
NDP	NDP, norrin cystine knot growth factor	3.697
CYLD	CYLD lysine 63 deubiquitinase	-0.024
DVL2	Dishevelled segment polarity protein 2	-0.592
DVL3	Dishevelled segment polarity protein 3	-0.370
DVL1	Dishevelled segment polarity protein 1	0.397
DKK1	Dickkopf WNT signaling pathway inhibitor 1	-2.906
BCL9	B cell CLL/lymphoma 9	-0.437
SENP2	SUMO specific peptidase 2	-0.050
CBY1	Chibby family member 1, beta catenin antagonist	-3.196
LEF1	Lymphoid enhancer binding factor 1	-0.481
SFRP1	Secreted frizzled related protein 1	-0.246

Table S3. Gene expression ratios in AMPK signaling pathway in GTE-treated OF zebrafish compared to those in OF control zebrafish.

Gene Symbol	Description	GTE vs. OF (log ratio)
AMPK	AMP-activated protein kinase	0.695
Ras GTPase	Ras GTPase	-10.316
PKA	cAMP dependent protein kinase	0.266
NF-kB family	Nuclear factor NF kappa B family	-0.465
calmodulin	Calmodulin	-1.263
CRY1	Cryptochrome circadian regulator 1	0.639
RAF1	Raf-1 proto-oncogene, serine/threonine kinase	-0.065
CAMKK2	Calcium/calmodulin dependent protein kinase kinase 2	-1.362
STK11IP	Serine/threonine kinase 11 interacting protein	-0.810
STK11	Serine/threonine kinase 11	-0.177
RASGRP1	RAS guanyl releasing protein 1	0.176
CAB39	Calcium binding protein 39	-0.622
MLST8	MTOR associated protein, LST8 homolog	0.288
TSC2	TSC complex subunit 2	-0.807
MAPK1	Mitogen-activated protein kinase 1	-0.132
PTGS2	Prostaglandin-endoperoxide synthase 2	-8.624
MAP2K2	Mitogen-activated protein kinase kinase 2	-0.195
RPTOR	Regulatory associated protein of MTOR complex 1	0.064
MAP2K1	Mitogen-activated protein kinase kinase 1	-0.021
FOXO1	Forkhead box O1	-0.200
ACACB	Acetyl-CoA carboxylase beta	-0.397
RASGRF1	Ras protein specific guanine nucleotide releasing factor 1	4.007
G6PC	Glucose-6-phosphatase catalytic subunit	0.725
ULK1	Unc-51 like autophagy activating kinase 1	-0.728
ATG13	Autophagy related 13	0.203
SIRT1	Sirtuin 1	-0.114
ACACA	Acetyl-CoA carboxylase alpha	0.347
FBXL3	F-box and leucine rich repeat protein 3	-0.066
MTOR	Mechanistic target of rapamycin kinase	-0.285
HMGCR	3-hydroxy-3-methylglutaryl-CoA reductase	-1.015
STRADA	STE20-related kinase adaptor alpha	-0.439
HDAC5	Histone deacetylase 5	-0.321
LIPE	Lipase E, hormone sensitive type	0.270
LONP1	Lon peptidase 1, mitochondrial	-1.300
RPS6KA1	Ribosomal protein S6 kinase A1	-0.473
SREBF1	Sterol regulatory element binding transcription factor 1	-0.716
TBC1D1	TBC1 domain family member 1	-1.032

Table S4. Details of feeding zebrafish.

Group name	Gluten granules (mg/fish/day)	Feeding frequency of Gluten granules (time/day)	Amount of *Artemia* at the 3rd week (mg cysts/fish/day)	Feeding frequency of *Artemia* (time/day)
NF	6	3	5	1
OF	6	3	60	3
OF + GTE	6	3	60	3

Table S5. Primer pair sequences, accession numbers and product sizes of the studied genes.

Gene Name	Accession NO.	Forward Primer	Primer	Product Size (bp)
gsk3b	NM_131381	GAAGCCATTGCCTTGTGCTC	TGACATTTGGTTCCCGCAGT	112
ctnnb1	NM_131059.2	GACAGGACGACCCAAGCTAC	GCCGTCTACGGGGTAATCA	128
prkaa1	NM_001110286.1	TGTGAGGACGCAGCAAAAGG	GAGGTAAGAGAAGAGGCCAG	98
prkaa2	XM_695739.8	CGTCAAGAAGGCAAAGTGGC	TTCTTCCGGCGCACTCTTAG	144
srebf1	NM_001105129	CAGAGGGTGGGCATGCTGGC	ATGTGACGGTGGTGCCGCTG	118
mmp2	NM_198067	TTGCTTCCCTGCAAACTTTTG	GAGCCACTTCTTTGTCTGTGTGA	87
sox9a	NM_131643	AATCTGAAGACGGCAGCGAA	GAGTGCACTTCTCCCATGCT	103
bact	AF057040	ATTGACTCAGGATGCGGAA	GAGGGCAAAGTGGTAAACG	123

Figure S1. GTE reduced VAT and decreased plasma TG and TCHO levels in an adult zebrafish independent replicate experiment.

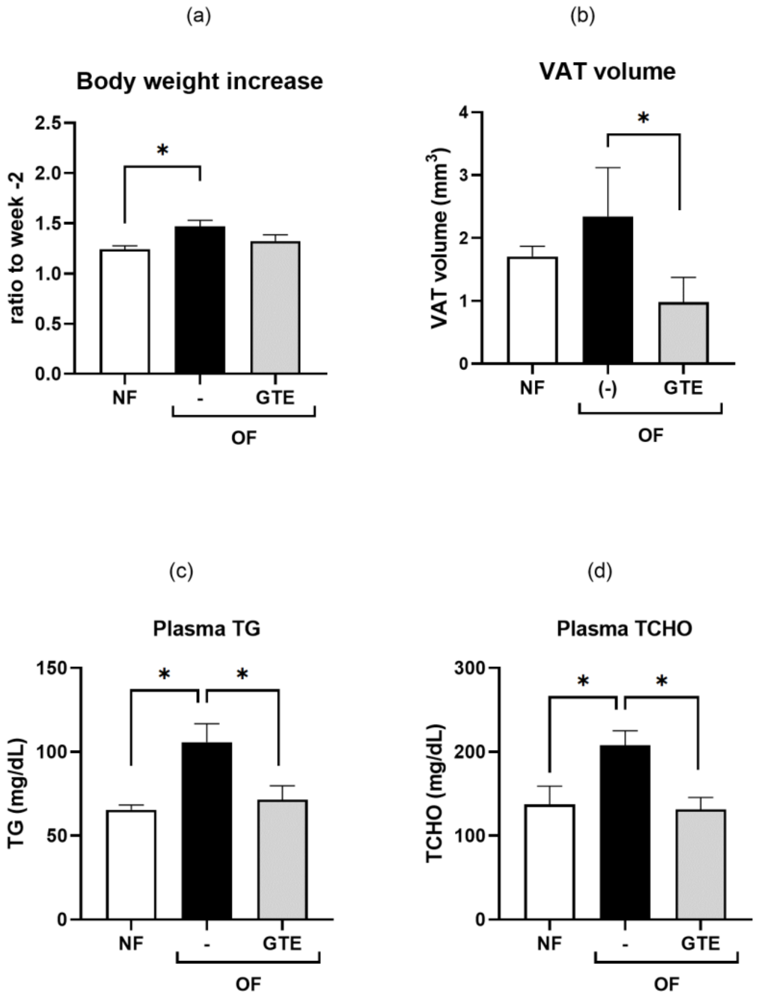

Supplement: Supplementary file 1 [file molecules-24-03256-s001.pdf]
